# *QuickStats*: Percentage* of Adults Aged ≥18 Years With or Without Psychological Distress^†^ Who Were Current Smokers,^§^ by Age Group and Level of Distress — National Health Interview Survey,^¶^ 2014–2016

**DOI:** 10.15585/mmwr.mm6723a6

**Published:** 2018-06-15

**Figure:**
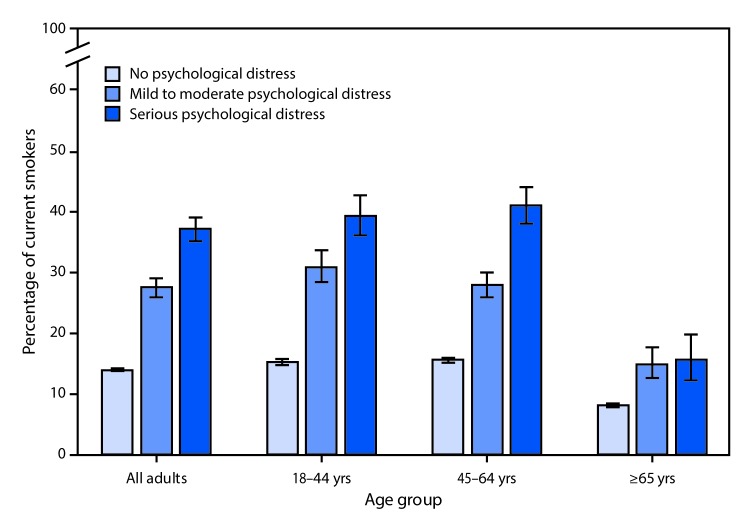
During 2014–2016, 37.2% of adults aged ≥18 years with serious psychological distress were current smokers, followed by 27.6% of those with mild to moderate psychological distress and 14.0% of those with no psychological distress. Among adults aged 18–44 and 45–64 years, the percentage of adults who were current smokers increased with the level of psychological distress. Among adults aged ≥65 years, the percentage who were current smokers was less among adults with no psychological distress than among adults with mild to moderate or serious psychological distress.

**Source:** National Health Interview Survey, 2014–2016. https://www.cdc.gov/nchs/nhis/index.htm.

